# Evaluation of Xpert MTB/RIF assay for the diagnosis of extrapulmonary tuberculosis in Southwest China

**DOI:** 10.1371/journal.pntd.0011403

**Published:** 2023-06-26

**Authors:** Tong-xin Li, Jing Wang, Yu-sheng Yang, Peng-sen Wang, Gang Zhou, Chuan-yu Liao, Hui-zheng Zhang, Ming Luo, Xiao-gang Zeng, Guo-qiang Yang, Li-jun Yang, Yao-kai Chen

**Affiliations:** 1 Central Laboratory, Chongqing Public Health Medical Center, Chongqing, China; 2 Clinical Laboratory, Chongqing Public Health Medical Center, Chongqing, China; 3 Department of Orthopaedics, Southwest Hospital, the Third Military Medical University, Chongqing, China; 4 Thoracic Surgery, Chongqing Public Health Medical Center, Chongqing, China; 5 General Surgery and Orthopedics Department, Chongqing Public Health Medical Center, Chongqing, China; 6 Department of infectious diseases, Chongqing Public Health Medical Center, Chongqing, China; Institut Pasteur, FRANCE

## Abstract

The purpose of this study was to determine the diagnostic efficacy of Xpert MTB/RIF assay for rapid diagnosis of ***Tuberculosis* (**TB) and detection of rifampicin (RIF) resistance in patients suspected of having EPTB, assessing it against traditional culture and drug susceptibility test (DST) by proportional method, and the ability to predict multidrug resistance TB by Xpert MTB/RIF assay. In this study, the Xpert MTB/RIF assay was applied to 1,614 extrapulmonary specimens. Compared with TB culture and Composite Reference Standard (CRS), the Xpert MTB/RIF assay had a high sensitivity and specificity for detection of EPTB. Depending on the culture method or CRS as the standard, sensitivity of the Xpert MTB/RIF assay for detection of MTB in pleural effusion, cerebrospinal fluid, thoracic drainage fluid and throat swabs specimens were lower than that of other specimens. According to the experimental results, we have reason to believe that Xpert MTB/RIF assay is a rapid and simple technique with high sensitivity and specificity for diagnosing EPTB and detecting drug resistance in variety of specimens. Xpert MTB/RIF assay combined with DST maybe identify more cases of multi-drug resistant tuberculosis (MDR-TB).

## Author summary

Tuberculosis (TB) is the second most deadly infectious disease after COVID-19. Improving the accessibility of WHO recommended rapid molecular diagnostic tests to achieve earlier and more accurate diagnosis of TB is an important part of strengthening TB laboratory work in the strategy of ending the epidemic of tuberculosis. Extrapulmonary tuberculosis (EPTB) accounts for 15% of all tuberculosis cases. Due to the unique biological samples of different classified EPTB and the occult nature of their onset, their diagnosis is more challenging. Smear microscopy is often insensitive and culture methods are prolonged. With the introduction of Xpert MTB/RIF assay (Cepheid Inc.), this has changed TB diagnostics by providing a rapid accessible platform to diagnose TB and identify rifampicin(RIF) resistance within 2h. Xpert MTB/RIF assay is a suite of semi-nested real-time fluorescent quantitative PCR in vitro diagnostic technologies with good detection efficiency. In recent years, WHO has limited recommended Xpert MTB/RIF assay to detect various types of samples other than sputum for preliminary diagnosis of EPTB. The purpose of this paper is to systematically evaluate the practical application effect of Xpert MTB/RIF assay in the clinical diagnosis of EPTB, and provide scientific guidance for clinicians.ű

## Introduction

According to WHO estimates, India (27.4%), China (8.9%), Indonesia (8.4%) and the Philippines (5.8%) account for about 50% of new TB cases worldwide, and the estimated number of new TB cases in China is 889,000 in 2018 [1]. Accurate and rapid diagnostic tests for TB are key to limiting the spread of the epidemic [1,2]. TB is clinically classified as pulmonary TB (PTB) and extrapulmonary TB (EPTB). The diagnosis of EPTB is more difficult and challenging than that of PTB. Existing tests, such as smears and cultures are limited by their lack of sensitivity and are time consuming (1~2 months) [3].

Xpert MTB/RIF assay(hereinafter referred to as “Xpert MTB/RIF”), is an automated nucleic acid amplification test designed for detecting a TB-specific *rpoB* gene fragment and can simultaneously detect rifampicin (RIF) resistance within 2 hours [4]. Since 2010, Xpert MTB/RIF has been recommended by the World Health Organization for the initial diagnosis of PTB [5], and for the diagnosis of EPTB since 2013 [6]. Compared with the mycobacterial culture, Xpert MTB/RIF is a better choice for the rapid (~2 hr) diagnosis of tuberculosis. However, the performance of Xpert MTB/RIF in the diagnosis of EPTB was found to be variable among different regions [7]. WHO has recommended Xpert MTB/RIF over the conventional tests (including conventional microscopy, culture or histopathology) for testing non-respiratory specimens (cerebrospinal fluid, pleural effusion, lymph nodes and other tissues) from patients suspected of having EPTB [8]. However, this was a conditional recommendation requiring more confirmatory evidence. This is an article based on the detection of extrapulmonary tuberculosis samples by Xpert MTB/RIF. Its sample types are relatively rich and the number is large. The aim of this study was to evaluate the application of the Xpert MTB/RIF in diagnosing EPTB and in detecting RIF-resistance, and to compare drug susceptibility test (DST) for screening of multi-drug resistant tuberculosis (MDR-TB) in Sounthwest China.

## Materials and methods

### Ethics statement

Ethics clearance for the use of specimens for laboratory evaluations was obtained from the Ethics Committee of Chongqing Public Health Medical Center(NO.2019-015-01-KY).

### Laboratory site and specimen receipt

The study was performed in Chongqing Public Health Medical Center, which provides routine diagnostic services to public sector hospitals in metropolitan Chongqing, and adjacent areas of *Sichuan*, *Guizhou* and *Hubei* provinces in SouthwestChina. The EPTB specimens were obtained from hospitalized patients. All specimens were collected aseptically by clinicians except urine and feces. The EPTB specimens submitted for routine mycobacterial culture and Xpert MTB/RIF from 23 July, 2014 to 31 March, 2017 were evaluated in this study and all cases were negative for HIV antibodies by serological test. This was solely a laboratory-based study, and no patient demographics were recorded other than those provided by the requesting clinician such as age, gender, and location. The reference strain of *Mycobacterium tuberculosis* (*H37Rv*) was obtained from the National Tuberculosis Reference Laboratory of the Chinese Center for Disease Control and Prevention.

### Mycobacterial culture of EPTB specimens

EPTB specimens from both children and adults were included in the study. All specimens were collected and handled according to the diagnosis of tuberculosis bacteriology inspection procedures of Chinese Antituberculosis Association [9]. Processed samples were inoculated into MGIT 960 non-radiometric automated isolation system [Becton Dickinson, Sparks, MD, USA]; the MGIT tube containing 7ml of 7H9 medium, supplemented with 0.8ml of Oleic Acid-Albumin-Dextrose-Catalase [OADC] along with Polymyxin B-Amphotericin B-Nalidixic acid- Trimethoprim-Azlocillin [PANTA], was inoculated with 0.5ml of decontaminated sample. The general steps of handling specimen are as follows: specimens were decontaminated using the N-acetyl-L-cysteine (NALC)–NaOH method [10] for 15 min, neutralized with sterile phosphate-buffered saline (PBS, pH 6.8), added to a final volume of 45 ml, and centrifuged at 3,000 *g* for 17 min at 4 *°C*. The supernatant was discarded, and the remaining pellet was resuspended in 1.0 ml of PBS. The suspension was divided into two parts; one used for the MGIT 960 tube and the other for neutral *Löwensteine-Jensen* (L-J) medium (Zhuhai Encode Medical Engineering Co., Ltd, Zhuhai, China). Each MGIT 960 tube was inoculated with 0.5 ml of the suspension, incubated at 37 *°C* in an automated MGIT 960 apparatus (Becton Dickinson) for a maximum of 42 days, and monitored continuously. The MGIT 960 outcomes were recorded as per the manufacturer’s instructions. Each L-J medium was inoculated with 0.1 ml of the resulting specimen, incubated at 37 *°C* in biochemical incubator for a maximum of 56 days, and observed manually. The culture was regarded as positive if one or both of the above two culture methods produced positive results.

### Mycobacterial drug susceptibility testing

All positive culture isolates were tested for drug susceptibility, which was determined by the method of proportional DST described by Canetti et al. [11], using the L-J medium with drugs [12]. Identification of M. tuberculosis was based on colony morphology and bio-chemical reactions [Thiophene-2-hydrazine carboxylate (TCH) and p-nitrobenzoic acid (PNB)]. The drugs and their critical concentrations for resistance were as follows: isoniazid(INH) 0.2 μg/mL, RIF 40 μg/mL, PNB 500μg/ml, TCH 5μg/ml. DST kit and M. tuberculosis identification kit were provided by Zhuhai Encode.

### Xpert MTB/RIF assay

The pretreatment of specimens was conducted and tested by Xpert MTB/RIF followed the manufacturer’s instructions (Cepheid, Sunnyvale, CA, USA). A 2:1 volume of sample reagent (SR) buffer was added to biopsy specimens after they had been chopped into very small pieces with a sterile blade in a sterile petri dish. Fluids larger than 2 ml were centrifuged at 3000rpm/min, the supernatant was decanted to remove the precipitate, and a 2:1 volume of SR buffer was added. 1 to 2 ml of fluids were added directly to a 2:1 volume of SR buffer. Fluids less than 1 ml were processed directly by the addition of a 2:1 volume of SR buffer, which was raised to 2 ml by the addition of SR buffer. The test results of Xpert MTB/RIF are automatically interpreted by the supporting software system. The MTB test results were divided into undetected (negative), extremely low (positive), low (positive), medium (positive) and high (positive). In case where results were reported as being “invalid,” “no result,” or “error,” the sample was reprocessed and rerun if sufficient material was available. The results of RIF resistance were divided into “detected”, “not detected” and “uncertainty”. If the MTB test results are “extremely low (positive)” or RIF resistance results are “uncertain”, neither was included in the statistical analysis.

### Data analysis

All data were entered into MS Excel and analyzed using SPSS v23. The culture result was considered the reference standard for sensitivity and specificity calculations for diagnosis of TB. Proportional method DST was considered the reference standard for RIF resistance. The CRS for this study was composed of smear microscopy, culture (both liquid and solid), clinical findings, histology/cytology, site specific computerized tomography scan/magnetic resonance imaging, and follow-up (FU) after 3 months from the date of enrollment [13]. These calculations were performed on the total sample as well as for individual specimen types. *Kappa* coefficient was used to measure the actual agreement between the two methods. A *kappa* value over 0.75 was considered excellent agreement, 0.40 to 0.75 as fair to good agreement, and below 0.40 as poor agreement. *p* < 0.05 was used to assess statistical significance. To compare the difference of paired data between Xpert MTB/RIF and gold standard, the *McNemar* test was used for correlated proportions. *McNemar* value >0.05 for the comparison of the two diagnosis methods was used to assess statistical consistency.

## Results

### Characteristics of the study population

**Fig 1:**
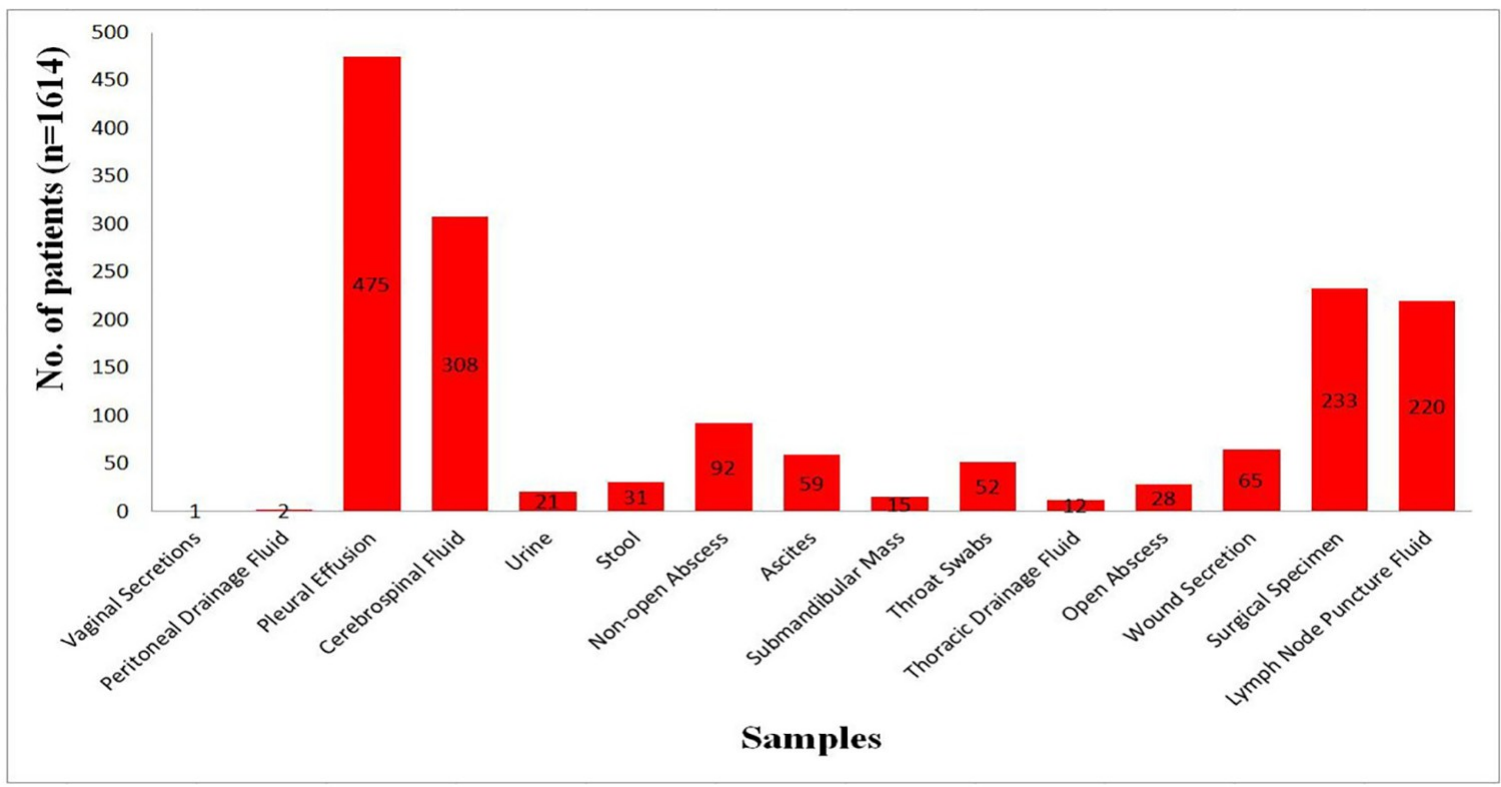
The details of 1614 samples.

A total of 1,614 specimens were from patients ranging in age from 10 to 87 years with a median age of 34.9 years, of whom 115 were younger than 18 years, 1,499 were older than 18 years, and 60.9% were male. Samples collected for the study included pleural effusion: 475, cerebrospinal Fluid [CSF]: 308, surgical specimen: 233, lymph node puncture fluid: 220, non-open abscess: 92, wound secretion: 65, ascites: 59, throat swabs: 52, the remaining sample of different types: less than 50. Among 297 culture positive isolates, 21 cases were identified as *nontuberculosis mycobacteria* (NTM) and excluded from following statistical analysis. The vaginal secretions and peritoneal drainage fluid were excluded due to the very small sample size. The Fig 1 displayed the details of 1,614 samples.

### Description of EPTB specimens and test of Xpert MTB/RIF

All EPTB specimens were received in the laboratory and categorized as listed in Fig 1. Pleural fluid accounted for 29.4% of samples, followed by 19.08% for cerebrospinal fluid and 14.44% for surgical specimens. The remaining samples comprised urine (1.3%), stool (1.92%), non-open abscess (5.7%), ascites (3.66%), submandibular mass puncture (0.93%), throat swabs (3.22%), thoracic drainage fluid (0.74%), open abscess (1.73%), wound secretion (4.03%) and lymph node puncture fluid (13.63%). All specimens were analyzed by the Xpert MTB/RIF test. The twenty-one cases identified as NTM were negative by Xpert MTB/RIF.

### Performance of Xpert MTB/RIF compared to culture

Table 1 describes the accuracy of Xpert MTB/RIF versus mycobacterial culture for the various sample types and among the 1,590 specimens. *M*. *tuberculosis* positivity determined by Xpert MTB/RIF was 38.1% (606/1,590) compared to 17.4% (276/1,590) by TB culture. The sensitivity and specificity of Xpert MTB/RIF versus TB culture were 80.7% and 70.9%, respectively. Table 1 shows that Xpert MTB/RIF had higher sensitivity and lower specificity as a rapid method for detection of non-open abscess, submandibular mass, wound secretion, surgical specimen and lymph node puncture fluid.

**Table 1:** Diagnostic performance of the Xpert MTB/RIF with respect to different specimen groups upon comparison with culture results.

Sample type	Culture	Xpert MTB/RIF		Sensitivity	Specificity	Positive Predictive	Negative Predictive	*Kappa*	*P*	*McNemar*
		+	-							
Pleural Effusion (n = 470)	+	39	15	72.2%	85.8%	39.8%	96.0%	0.4285	<0.001	<0.001
	-	59	357							
Cerebrospinal Fluid (n = 308)	+	37	22	62.7%	96.8%	82.2%	91.6%	0.6542	<0.001	0.016
	-	8	241							
Urine (n = 21)	+	3	0	100%	83.3%	50.0%	100%	0.5882	0.003	0.25
	-	3	15							
Stool (n = 31)	+	1	0	100%	63.3%	8.3%	100%	0.1003	0.201	0.001
	-	11	19							
Non-open Abscess (n = 90)	+	16	2	88.9%	33.3%	25.0%	92.3%	0.114	0.063	<0.001
	-	48	24							
Ascites (n = 59)	+	1	0	100%	91.4%	16.7%	100%	0.2643	0.003	0.063
	-	5	53							
Submandibular Mass (n = 15)	+	3	0	100%	41.7%	30.0%	100%	0.2222	0.171	0.016
	-	7	5							
Throat Swabs (n = 52)	+	1	0	100%	88.2%	14.3%	100%	0.2239	0.01	0.031
	-	6	45							
Thoracic Drainage Fluid (n = 11)	+	1	1	50.0%	88.9%	50.0%	88.9%	0.3889	0.197	1
	-	1	8							
Open Abscess (n = 24)	+	6	0	100%	50.0%	40.0%	100%	0.3333	0.028	0.004
	-	9	9							
Wound Secretion (n = 61)	+	12	1	92.3%	33.3%	27.3%	94.1%	0.1372	0.067	<0.001
	-	32	16							
Surgical Specimen (n = 231)	+	58	2	96.7%	34.5%	34.1%	96.7%	0.1954	<0.001	<0.001
	-	112	59							
Lymph Node Puncture Fluid (n = 217)	+	44	10	81.5%	49.7%	34.9%	89.0%	0.2156	<0.001	<0.001
	-	82	81							

### Performance of Xpert MTB/RIF compared to CRS

Compared with the CRS (Table 2), *M*. *tuberculosis* positivity determined by Xpert MTB/RIF was 38.1% (605/1,590) compared to 60.9% (969/1,590). The sensitivity and specificity of Xpert MTB/RIF versus clinic diagnosis were 61.7% and 98.9%, respectively.

**Table 2:** Diagnostic performance of the Xpert MTB/RIF with respect to different specimen groups upon comparison with CRS.

Sample type	CRS	Xpert MTB/RIF		Sensitivity	Specificity	Positive Predictive	Negative Predictive	*Kappa*	*P*	*McNemar*
		+	-							
Pleural Effusion (n = 470)	+	95	116	45.0%	98.8%	96.9%	68.8%	0.4616	<0.001	<0.001
	-	3	256							
Cerebrospinal Fluid (n = 308)	+	45	71	38.8%	100%	100%	73.0%	0.4414	<0.001	<0.001
	-	0	192							
Urine (n = 21)	+	6	3	66.7%	100%	100%	80.0%	0.6957	0.001	0.25
	-	0	12							
Stool (n = 31)	+	11	1	91.7%	94.7%	91.7%	94.7%	0.864	<0.001	1
	-	1	18							
Non-open Abscess (n = 90)	+	64	6	91.4%	100%	100%	76.9%	0.826	<0.001	0.031
	-	0	20							
Ascites (n = 59)	+	6	0	100%	100%	100%	100%	1	<0.001	1
	-	0	53							
Submandibular Mass (n = 15)	+	10	0	100%	100%	100%	100%	1	<0.001	1
	-	0	5							
Throat Swabs (n = 52)	+	7	43	14.0%	100%	100%	4.4%	0.0124	0.569	<0.001
	-	0	2							
Thoracic Drainage Fluid (n = 11)	+	2	3	40.0%	100%	100%	66.7%	0.4211	0.087	0.25
	-	0	6							
Open Abscess (n = 24)	+	15	2	88.2%	100%	100%	77.8%	0.814	<0.001	0.5
	-	0	7							
Wound Secretion (n = 61)	+	42	5	89.4%	85.7%	95.5%	70.6%	0.6982	<0.001	0.453
	-	2	12							
Surgical Specimen (n = 231)	+	169	46	78.6%	93.8%	99.4%	24.6%	0.3144	<0.001	<0.001
	-	1	15							
Lymph Node Puncture Fluid (n = 217)	+	126	75	62.7%	100%	100%	17.6%	0.1986	<0.001	<0.001
	-	0	16							

Xpert MTB/RIF had the highest sensitivity on ascites and submandibular mass (100%), followed by stool (91.67%), wound secretion (89.36%) and open abscess (88.24%), as compared to the results of clinic diagnosis. The specificity values for Xpert compared to the CRS were lowest for wound secretion (85.7%), and surgical specimens (93.8%). Among the specimens, Xpert MTB/RIF was highly consistent with culture for ascites (*Kappa* = 1, *p* < 0.001; *McNemar* = 1), submandibular mass (*Kappa* = 1, *p* < 0.001; *McNemar* = 1), stoll (*Kappa* = 0.864, *p* < 0.001; *McNemar* = 1), open abscess (*Kappa* = 0.814, *p* < 0.001; *McNemar* = 0.5), wound secretion (*Kappa* = 0.6982, *p* < 0.001; *McNemar* = 0.453), and urine (*Kappa* = 0.6957, *p* = 0.001; *McNemar* = 0.25). The sensitivity and specificity of Xpert MTB/RIF for ascites and submandibular mass were 100%, while the specificity for urine and open abscess were 100%. These results suggest that Xpert MTB/RIF could have good diagnostic potential when applied as a rapid method for detection of ascites, submandibular mass, stool, open abscess, wound secretion and urine.

### Performance of Xpert MTB/RIF compared to DST

Furthermore, we analyzed drug resistance among TB positive specimens (222 specimens) using both culture and Xpert MTB/RIF (Table 3). The sensitivity and specificity of Xpert MTB/RIF versus Proportional method DST were 89.5% and 97.6%, respectively. Compared with traditional methods, Xpert MTB/RIF had a high sensitivity for pleural effusion, non-open abscess, thoracic drainage fluid, open abscess, wound secretion, surgical specimen, cerebrospinal fluid, and lymph node puncture fluid. The specificity values for Xpert MTB/RIF compared to Proportional method DST were lowest for wound secretion (90.0%) and surgical specimen (95.5%). In total, Xpert MTB/RIF was highly consistent with culture results (*Kappa* = 0.8806, *p* < 0.001; *McNemar* = 0.754). This suggests that the diagnosis of RIF resistance by Xpert MTB/RIF may be a reliable and rapid method for identifying RIF resistance of extrapulmonary tuberculosis patients.

**Table 3:** Sensitivities and specificities of the Xpert MTB/RIF for detection of RIF resistance compared with DST.

Sample type	Xpert MTB/RIF	DST		Sensitivity	Specificity	Positive Predictive	Negative Predictive	*Kappa*	*P*	*McNemar*
		+	-							
Pleural Effusion	+	3	0	100%	100%	100%	100%	1	<0.001	1
	-	0	36							
Cerebrospinal Fluid	+	19	0	86.4%	100%	100%	83.3%	0.837	<0.001	0.25
	-	3	15							
Urine	+	0	0	/	100%	/	100%	/	/	/
	-	0	3							
Stool	+	0	0	/	100%	/	100%	/	/	/
	-	0	1							
Non-open Abscess	+	3	0	100%	100%	100%	100%	1	<0.001	1
	-	0	13							
Ascites	+	0	0	/	100%	/	100%	/	/	/
	-	0	1							
Submandibular Mass	+	0	0	/	100%	/	100%	/	/	/
	-	0	3							
Throat Swabs	+	0	0	/	100%	/	100%	/	/	/
	-	0	1							
Thoracic Drainage Fluid	+	1	0	100%	/	100%	/	/	/	/
	-	0	0							
Open Abscess	+	1	0	100%	100%	100%	100%	1	0.014	1
	-	0	5							
Wound Secretion	+	0	1	100%	90.0%	66.7%	100%	0.75	0.007	1
	-	0	9							
Surgical Specimen	+	13	2	92.9%	95.5%	86.7%	97.7%	0.8621	<0.001	1
	-	1	42							
Lymph Node Puncture Fluid	+	9	1	81.8%	97.0%	90.0%	94.1%	0.8125	<0.001	1
	-	2	32							

### Summary of Xpert MTB/RIF compared to DST in MDR-TB

To explore the relationship of RIF resistant TB with INH resistant TB, we analyzed RIF results by Xpert MTB/RIF and INH resistant results in RIF resistant specimens (57 specimens) of the phenotypic DST (Table 4). There were 21 cases of INH resistant specimens out of 51 cases of RIF resistant specimens by Xpert MTB/RIF. In contrast, only 4 cases were INH resistant among the 6 cases of RIF negative specimens by Xpert MTB/RIF. Of the 57 RIF resistant specimens tested by DST, 25 cases (43.9%) were MDR-TB, accounting for 11.3% of the 222 culture-positive specimens. These results suggest that Xpert MTB/RIF combined with DST maybe identify more cases of MDR-TB in the EPTB.

**Table 4:** The results of RIF and INH resistance in cultured positive specime.

Specimen type	RIF (Xpert MTB/RIF)	DST	
		RFP Resistance	INH Resistance
Pleural Effusion	Positive	3	1
	Negative	0	0
Cerebrospinal Fluid	Positive	19	10
	Negative	3	1
Non-open Abscess	Positive	3	2
	Negative	0	0
Thoracic Drainage Fluid	Positive	1	0
	Negative	0	0
Open Abscess	Positive	1	0
	Negative	0	0
Wound Secretion	Positive	2	1
	Negative	0	0
Surgical Specimen	Positive	13	4
	Negative	1	1
Lymph Node Puncture Fluid	Positive	9	3
	Negative	2	2
Total	Positive	51	21
	Negative	6	4

## Discussion

Diagnosis of EPTB remains a challenge due to a lack of sensitive conventional laboratory techniques. Therefore, Xpert MTB/RIF nucleic acid amplification techniques may play an important role in rapid and accurate diagnosis. In this large screening analysis, we found that the positive predictive value and specificity of Xpert MTB/RIF for the diagnosis of EPTB were both substantially higher than previously recognized, especially for RIF resistance detection. This finding had important implications for the potential use of Xpert MTB/RIF, or similar tests, for EPTB screening.

Tuberculin Skin Test(TST) and Interferon Gamma Release assay(IGRA) are commonly used in vitro diagnostic methods based on immunology to assist in the diagnosis of tuberculosis or determine whether the body is infected with MTB.

A meta-analysis evaluated the predictive value of IGRA and nodal TST for the progression of latent infection to active tuberculosis [14]. The results show that IGRA has better predictive ability, and IGRA positive individuals may benefit from preventive treatment, but TST positive individuals may no. Double testing may improve the detection effect, but it needs further confirmation. In a word, TST is simple and economical, but its specificity is poor. It crosses with many NTMs in antigenicity, and the false positive is high. IGRA has high specificity and is less affected by NTM. Its positive results have certain significance for the diagnosis of tuberculosis, and the possibility of excluding tuberculosis from the negative results is high. The defect of IGRA is that *Mycobacterium Kansas*, *Mycobacterium stephensi* and *Mycobacterium Gordon* are prone to false positive results. At the same time, its sensitivity needs to be improved, especially in children and HIV infected people with immune deficiency.

China has reported the results of its first ever nationwide drug resistance survey, with documented proportions of MDR-TB of 5.7% among new cases and 25.6% among those previously treated, and this survey confirms previous estimates that about 100,000 MDR-TB cases are emerging in China annually [15]. It’s very important for quick and effective diagnosis of these cases. We found that it had a sensitivity of 61.7% (598/969) in CRS-positive patients, and the specificity for urine, ascites, submandibular mass, and open abscess were 100%. What’s more, the sensitivity and specificity for RIF resistance detection were 89.5% and 97.6%, respectively. We evaluated this technology for rapid and accurate diagnosis of EPTB, often a diagnostic dilemma.

To the best of our knowledge, there have been few studies evaluating Xpert MTB/RIF assay on non-respiratory samples in Southeast China comparing its efficacy against both solid and liquid culture. A study from South Africa found a marginally lower pooled sensitivity of 59% and a similar specificity of 92% compared to our study [16]. Studies from Turkey and Germany also had lower pooled sensitivity but substantially higher specificity ranging from 67.7% to 77.2% and 96.2% to 98.4%, respectively [17,18].

Compared with the culture results, the sensitivity results of the following samples detected by Xpert MTB/RIF in our research: The sensitivity of thoracic drainage fluid was 50.00%, cerebrospin fluid was 62.71%, and plural effect was 72.22%. The sensitivity of other samples was higher than 80%. The specificity of non-open abscess and wound secretion, surgical specimen, lymph node puncture fluid, and open Abscess were less than or equal to 50.00%. The probability of identifying NTM in these types of samples was higher than that in others, indicating that these patients were more likely to be infected with NTM. Among them, cerebrospinal fluid had the highest specificity, 96.8%, indicating that it is more likely to infect MTB in the central nervous system of tuberculosis hospital. Compared with CRS, the sensitivity of the following samples detected by Xpert MTB/RIF: pleural effusion was 45.02%, thoracic drainage fluid was 40.00%, cerebrospinal fluid was 38.79%, throat swabs was 14%, and the sensitivity of other samples was higher than 60%; The specificity results showed that the specificity of the wound secretion was 85.7%, and the other samples were more than 90.00%. This suggests that pleural effusion, thoracic drainage fluid, cerebrospinal fluid have limited diagnosis of tuberculosis, especially throat swabs. Although culture is the gold standard for the diagnosis of EPTB, it is thought to be not perfect, possibly due to the paucibacillary nature of the disease [19]. Therefore, it can lead to a false diagnosis of specimens as false positives by the Xpert MTB/RIF. The use of a composite gold standard classifies TB based on a positive result in one of several criteria, include conventional culture, clinical, histology, radiology or a response to treatment, resulting in a reclassification of these false positives to true positives and an increased specificity, although the CRS could lead to poor specificity, which is a limitation of the CRS [20].

Similar to earlier reports, we found a low sensitivity and high specificity on fluid specimens in the diagnosis of TB by Xpert MTB/RIF, compared with culture as the gold standard. Specifically, previous reports on pleural fluids had sensitivity ranging from 15% to 76%, and high specificity up to 100%, some of the poorer results being due to a small sample size [13,21–23]. When compared with the CRS, as explained previously, the sensitivity was reduced obviously, while the specificity was 100%. The poor sensitivity of Xpert MTB/RIF in fluids is thought to be due to interference by inhibitors such as blood, salts, proteins or cellular debris [24,25]. These interfere with the amplification enzyme and thereby inhibit the PCR [26].

In comparison with other studies, we evaluated Xpert MTB/RIF assay on a wide variety of specimens; however, the sample size for certain specimen types like thoracic drainage fluid and submandibular mass was limited. This finding also had important implications for the potential use of Xpert MTB/RIF, or similar tests, for EPTB and RIF resistance diagnosis. For a limited number of samples, whether the improved diagnostic lead-time reduces tuberculosis transmission or improves clinical outcome requires further study, Xpert MTB/RIF is still an expensive tool for most high burden countries, and the cost-effectiveness of this finding strategy needs to be further assessed. Our study will provide more data for future planned analyses.

### Limitation

First, some kinds of samples size were too small and excluded from research. Second, this study lack results of patients’ follow-up after treatment. Third, this research focused only RIF resistance.

### Ethics statement

These studies involving human participants were reviewed and approved in writing by the Ethics Committee of Chongqing Public Health Medical Center. Our study was conducted in accordance with the Declaration of Helsinki. The institutional review board of Chongqing Public Health Medical Center approved this study and waived the requirement for written informed consent. The institutional review board waived the need for informed consent because all patients’data were analyzed in anonymity and no additional informed consent was required.
